# PinX1: a sought-after major tumor suppressor at human chromosome 8p23

**DOI:** 10.18632/oncotarget.339

**Published:** 2011-10-20

**Authors:** Xiao Zhen Zhou

**Affiliations:** ^1^Department of Medicine, Beth Israel Deaconess Medical Center, Harvard Medical School, 330 Brookline Avenue, CLS 0408, Boston, MA 02215

**Keywords:** Tumor suppressor, telomere, telomerase, telomerase inhibitor, PinX1, chromosome instabilty, chromosome 8p23, LOH

## Abstract

Human chromosome 8p23 is a region that has the most frequent heterozygosity in common human adult epithelial malignancies, but its major tumor suppressor gene(s) remain to be identified. Telomerase is activated in most human cancers and is critical for cancer cell growth. However, little is known about the significance of telomerase activation in chromosome instability and cancer initiation. The gene encoding the potent and highly conserved endogenous telomerase inhibitor PinX1 is located at human chromosome 8p23. However, the role of PinX1 in telomerase regulation and cancer development is not clear. Recent works from our group indicate that PinX1 is critical for maintaining telomere length at the optimal length. Furthermore, PinX1 is reduced in a large subset of human breast cancer tissues and cells. Significantly, PinX1 inhibition activates telomerase, and elongates telomeres, eventually leading to chromosome instability, all of which are abrogated by telomerase knockdown or knockout. Moreover, PinX1 allele loss causes majority of mice to develop a variety of epithelial cancers, which display chromosome instability and recapitulate to 8p23 allele loss in humans. These results indicate that PinX1 is a sought-after major tumor suppressor at human chromosome 8p23 that is essential for regulating telomerase activity and maintaining chromosome stability. These results suggest that inhibition of telomerase using PinX1 especially its telomerase inhibitory fragment or other methods might be used to treat cancers that have telomerase activation.

## CHROMOSOME 8P23 AND HUMAN CANCERS

Inactivation of tumor suppressor genes due to gene alterations, notably loss of heterozygosity (LOH), plays a major role in the development of common human adult cancers [1, 2], with breast cancer as a good example [3-8]. Chromosome 8p23 is one of the most frequent LOH regions in common human adult epithelial malignancies, including breast, liver, lung, and gastrointestinal cancers [9-26]. For example, up to 70% of hepatocellular carcinomas [9-14] and 60% of human gastric cancer [26] exhibit LOH at 8p23 near the maker D8S277. 8p23 is also a common integration site for hepatitis B virus (HBV), a well-known major risk factor in liver cancer [10, 27]. Similarly, LOH on 8p is found in up to 50% of breast carcinomas and is often associated with advanced tumor stage and aggressive histology [15-19]. Although several potential tumor suppressors have been mapped to this region, including Nkx3.1 at 8p21 and FEZ1/LZTS1 at 8p22 [21, 28-30], even the combined rates of loss of these genes could not account for the extensive alterations seen in human tumors [21], indicating that major tumor suppressor gene(s) remain to be identified.

## TELOMERASE ACTIVATION AND CANCER

Maintaining optimal telomere length is crucial for cells since excessive telomere loss leads to telomere fusions, anaphase bridges, aberrant mitotic chromosome separation and aneuploidy [31-34]. Significantly, these telomere dysfunction phenotypes are very common in human cancer, especially epithelial cancers [34-38]. Moreover, long-term lack of telomerase in mice leads to telomere shortening and dysfunction, and promotes oncogenesis in a certain condition such as lack of tumor suppressors p53 [34, 39-43] or DNA mismatch repair protein MSH2 [44] or in the presence of telomeric repeat binding factor TRF2 overexpression [45], and also shifts the tumor spectrum of p53 mutant mice to include more epithelial cancers [42]. Thus, telomere dysfunction caused by excessive telomere loss has been proposed as a major cause of chromosome instability in epithelial carcinogenesis [35, 38, 42].

However, it has been well documented that telomere shortening due to knockout of telomerase activates DNA damage pathways, limits cell proliferation and inhibits tumorigenesis in a number of mouse models [46-50]. Moreover, although telomere loss in cancers has well documented, telomere elongation is quite common in a variety of epithelial cancers, e.g. in >40% of liver cancer [51], esophageal cancer [52] and brain tumors [53], and also correlates with advanced stages and/or poor survival in some cancers [51, 52, 54-57]. Even longer telomeres in blood cells are shown to be associated with higher cancer risk and poor survival in breast cancer [58]. However, little is known about the consequence of aberrant telomere elongation.

**Figure 1:**
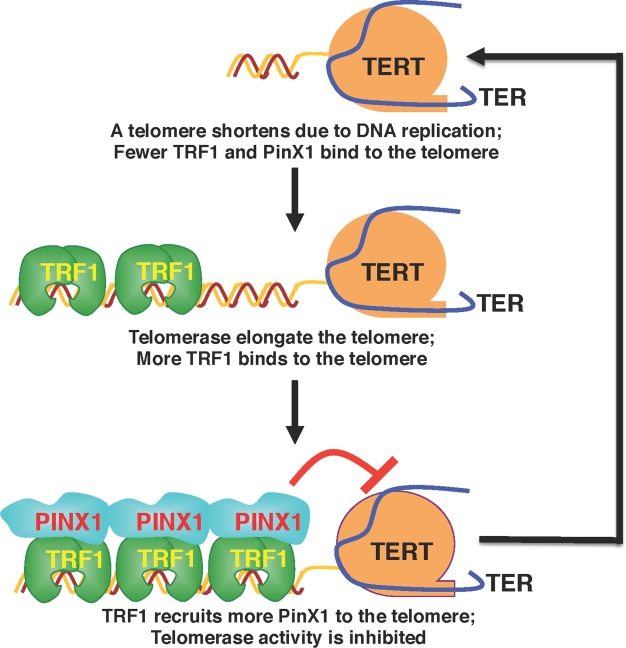
The role of PinX1 in maintaining telomeres at the optimal length When telomerase extends a telomere to a certain length, the elongated telomere binds more TRF1, which might in turn recruit more PinX1 to the telomere. Once concentrated locally on telomeres at a high concentration, simple mass-action might allow PinX1 to more effectively stop telomerase from adding more repeats. Conversely, when a telomere is shortened following each cell division due to the end replication problem, the shortened telomere contains less TRF1, which might recruit fewer PinX1 or none at all to the telomere. Therefore, the telomere might have a greater chance of being elongated due to telomerase being inhibited less, or even not at all. This Pinx1-mediated negative feedback mechanism might help maintain telomeres at a similar median length. (Adapted from Soohoo,et al., 2011, J Biol Chem 286:3894-3906).

In mammalian cells, telomere surveillance is carried out in cis by shelterins, which include three telomeric DNA-binding proteins TRF1, TRF2 and POT1, and their associating proteins [59]. While TRF2 [32, 60] and POT1 [60-62] protect telomere integrity, TRF1 maintains telomeres at the optimal length [32, 59, 63]. Indeed, many TRF1-interacting or associated proteins influence telomere length [63-67]. We identified TRF1 as Pin2 in the same screen for Pin1 [68, 69]. Both Pin1 and Pin2/TRF1 are important for mitotic regulation [69-73] and Pin1 directly regulates TRF1 function in telomere maintenance and aging [74]. By identifying new TRF1/Pin2-interacting proteins, including PinX1-4, we have further identified important TRF1 function in telomere maintenance and mitosis [66, 74-76].

## PINX1 IS A TELOMERASE INHIBITOR ESSENTIAL FOR MAINTAINING TELOMERASE ACTIVITY AND TELOMERE LENGTH

Unlike other TRF1-interacting proteins, PinX1 has a unique property to directly bind to TERT and TERC and inhibit telomerase activity at telomere [66, 77] . Furthermore, PinX1 inhibition in cancer cells activates telomerase and elongates telomeres, whereas PinX1 overexpression has the opposite effects [66]. The ability of PinX1 to regulate telomerase and telomere length is also conserved in yeast, rats and fish [78-80]. In fact, yeast PinX1 has been shown to inhibit telomerase by sequestering its protein catalytic subunit in an inactive complex lacking telomerase RNA in the nucleolus [78]. However, it was not clear why such telomerase inhibitor is needed until recently [81, 82].

By performing the extensive structure-function analysis to define the PinX1-TRF1 interaction and to dissect the biological function of the PinX1-TRF1 interaction in regulating telomere maintenance, we have demonstrated that the TRF1-PinX1 interaction is required not only for targeting PinX1 to telomeres, but also for PinX1 to prevent abnormal telomere elongation in cells [81]. Given the unique and potent ability of PinX1 to inhibit telomerase activity and telomere elongation in cells [66, 83], our results suggest that the TRF1-PinX1 interaction affects the loading of PinX1 onto telomeres to prevent telomere elongation [81]. This may provide an additional level of telomerase regulation, in conjunction to the physical occlusion from telomeres by Pot1 [67, 84], by supplying a link between TRF1 and telomerase inhibition that contributes toward maintaining telomere homeostasis (Fig. 1). TRF1 binds along the duplex part of the telomere and functions as a measuring device to assess telomere length [59, 63, 85, 86]. For telomere length homeostasis to be effective, the information about the length of the telomere needs to be relayed from TRF1 to telomerase via other proteins since TRF1 does not directly affect telomerase activity [59, 63]. Our data indicate that the telomerase inhibitor PinX1 might be recruited by TRF1 to a telomere to stop telomerase action as the telomere is being elongated and reaches a certain threshold [81].

This model (Fig. 1) has been recently supported by manipulating PinX1 expression in vitro and in vivo to demonstrate that PinX1 is a rate-limiting physiological regulator of telomerase and telomere length [82]. We have shown that reduced PinX1 expression activates telomerase and leads to telomere lengthening and chromosome instability, which can be fully rescued by TERT knockdown or knockout [82], confirming that PinX1 acts as a telomerase inhibitor to regulate telomere maintenance. The significance of these results is demonstrated by our observations that most PinX1+/- mice develop aggressive cancers that display telomere lengthening and chromosome instability [82]. These results provide the first evidence for an essential role of PinX1 in regulating telomerase activity and telomere length [82].

## PINX1 IS A SOUGHT-AFTER MAJOR TUMOR SUPPRESSOR AT 8P23 ESSENTIAL FOR CHROMOSOME STABILITY

Notably, PinX1 gene localizes to human chromosome 8p23 [13, 66], which is one of the regions that experiences the most frequent loss of heterozygosity (LOH) in many common human cancers [9, 10, 12, 15, 19, 21, 25, 26]. For example, 8p23 LOH has been found in up to 70% of hepatocellular carcinomas [9, 10, 12, 13], 60% of gastric cancer [26], and 50% of breast cancer [15, 19]. This region is also a common integration site for hepatitis B virus, a well-known major risk factor in liver cancer [10] and PinX1 is reduced in ~ 40% hepatitis B virus-related liver cancer [13]. Moreover, PinX1 inhibition potently increases, whereas PinX1 overexpression suppresses, tumorigenicity of cancer cells [66]. Thus, PinX1 might be a putative tumor suppressor. Subsequent PinX1 studies on human cancer samples are inconclusive [26, 87]. Moreover, there is no genetic evidence for any involvement of PinX1 in cancer.

The recent results from my laboratory indicate that PinX1 is a major tumor suppressor at 8p23 [82]. PinX1 is downregulated in a large subset of human breast cancer tissues and in most breast cancer cell lines examined [82]. PinX1 expression is gene dosage-dependent; ablation of one allele reduces protein level by 60-70% in vitro and in vivo. Importantly, reducing PinX1 by gene knockout or knockdown not only increases telomerase activity and telomere length, but also leads to chromosome instability in cell models [82]. These telomere-related phenotypes are fully reversed by knockout or knockdown of telomerase. Moreover, telomere lengthening induced by PinX1 inhibition occurs at most chromosome ends, a feature of cancer cells [88]. The significance of these findings is further substantiated by the demonstrations that nearly all PinX1+/- mice develop a range of epithelial malignancies with evidence of telomere lengthening and chromosome instability.

**Figure 2:**
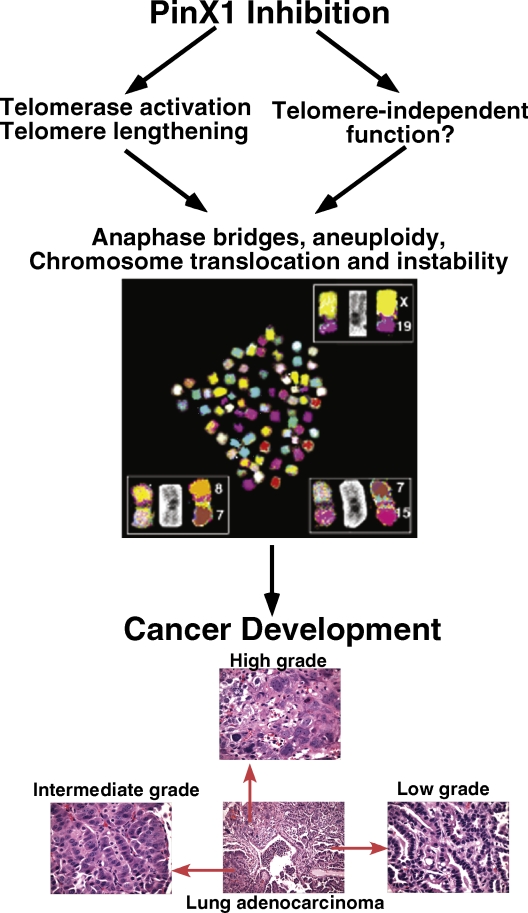
PinX1 as a sought-after major haploinsufficient tumor suppressor at 8p23 that is essential for chromosome stability The human PinX1 gene is located at 8p23, a frequent LOH in common human epithelial cancers and its protein expression is often down-regulated in many human cancer tissues and cell lines. While PinX1 null mice or cells are lethal, reducing PinX1 expression via heterozygous knockout or knockdown increases telomerase activity and leads to telomere lengthening, anaphase bridges, chromosome translocation and instability, as shown in the picture. Moreover, PinX1 heterozygous knockout causes most mice to spontaneously develop a range of malignant tumors displaying evidence of telomere lengthening and chromosomal instability. Notably, the majority of cancers in the PinX1 mutant mice are carcinomas and share tissues of origin with human cancer types linked to 8p23 alterations. Furthermore, many PinX1 heterozygous knockout mice develop more than one type of tumor and diverse histopathologies are observed in the same types of tumors among different mice or even in the same tumors, as shown for lung cancer. (Adapted from Zhou,et al., 2011, *J Clin Invest* 121:1266-1282)

Although most human cancers are epithelial carcinomas, common tumor suppressor mutant mice mainly develop lymphomas and soft tissue sarcomas, with a very few exceptions [89, 90]. Notably, most tumors in PinX1+/- mice are epithelial carcinomas arising in organs that are known to develop common cancers in humans, including lung, mammary, liver and gastrointestinal tract cancers [82], which are also known to have frequent LOH at 8p23 in humans [9, 10, 12, 20-22, 24, 25]. Most tumors showed features commonly seen in advanced human carcinomas such as nuclear atypia, desmoplasia, stromal invasion and/or lung metastasis [82]. Moreover, 20% of PinX1+/- mice developed two or three cancer types within the same animals. Even within one tumor type, there were diverse histopathologies in different mice, the same mice or even within the same tumors [82]. Thus, almost all PinX1+/- mice spontaneously develop a range of aggressive epithelial cancers [82], which are unusual in mice, even after deleting many other tumor suppressors [89, 90], but are known to have 8p23 LOH in humans. These results suggest that PinX1+/- cancers likely originate from multiple cells and behave aggressively. Given activation of telomerase in most human cancers and common downregulation of PinX1 in liver, gastric and breast cancers [13, 26, 82], these results indicate that PinX1 is a major tumor suppressor, whose downregulation activates telomerase, induces chromosome instability and eventually leads to tumorigenesis by [82].

## HOW DOES PINX1 REGULATE CHROMOSOME STABILITY AND TUMORIGENESIS?

At the present time, we do not yet know the molecular mechanism by which reducing PinX1 function leads to chromosome instability and tumorigenesis. Notably, we have shown that the PinX1 knockout phenotypes including telomerase activation, telomere elongation, anaphase bridges, aneuploidy and chromosome instability are fully suppressed by knockdown or knockout of TERT or TERC, indicating that telomerase is essential for PinX1 reduction to induce chromosome instability [82]. Moreover, it takes time for PinX1-induced telomerase activation to induce telomere elongation and chromosome instability when PinX1 is knocked out or down [82]. Notably, the PinX1 and p53 double mutant mice have similar tumor spectrum [82] that are found in TERC and p53 double mutant mice due to telomere loss [42] or in TPP1/ACD and p53 double mutant mice due to telomere deprotection [91]. Furthermore, abnormal telomere elongation is common and also correlates with advanced stages and/or poor survival in some cancers [51, 52, 54, 57]. Moreover, TERC is required for the tumor-promoting effects of TERT overexpression in transgenic mice [92]. These results together suggest that abnormal telomerase activation and telomere lengthening due to loss of PinX1 might have similar effects on the development of epithelial cancers, as does telomere shortening or telomere deprotection. Given that PinX1 directly binds to and inhibits TERT [66] and is targeted by TRF1 to telomeres to prevent abnormal telomere elongation by telomerase [93], it is conceivable that when PinX1 is inhibited, telomerase is aberrantly activated without a proper brake and eventually leads to chromosome instability possibly via inducing aberrant telomere elongation to compromise telomere function [82]. Consistent with this idea is the previous findings that loss of p80/p95 in *Tetrahymena* induces telomere lengthening and chromosome instability [94]. However, telomerase has other telomere-independent function such as in DNA damage response [95, 96] and activating β-catenin [97]. Similarly, PinX1 might have non-telomeric functions such as in RNA maturation [98] and cell division [99]. Moreover, it has been shown that TERT overexpression extends life span in normal human cells in vitro [100-102] and that short-term telomerase reactivation reverses tissue degeneration in aged telomerase-deficient mice [103], although long-term TERT overexpression promotes tumorigenesis in vitro or in vivo [92, 104-110]. These results suggest that long-term loss of PinX1 function and/or telomerase overexpression might affect other cellular processes that contribute to chromosome instability and tumorigenesis. Therefore, further experiments are needed to define how PinX1 controls chromosome stability via telomere-dependent and/or -independent telomerase and/or other mechanisms unrelated to telomerase [82].

Although it is not clear how PinX1 downregulation leads to chromosome instability, we have demonstrated that PinX1-induced chromosome instability plays a major and novel role in tumorigenesis [82]. We show that PinX1 is reduced in most human breast cancer tissues and cells and that reducing PinX1 levels leads to telomerase activation, telomere elongation and chromosome instability [82]. Moreover, almost all PinX1 heterozygous knockout mice develop a range of epithelial malignancies, with multiple tumor types in the same mice, diverse cell morphologies/grades in one tumor type among mice, or even within individual tumors [82]. PinX1 heterozygous knockout also shifts the p53 mutant tumor spectrum to epithelial carcinomas [82]. Importantly, PinX1+/- cancer cells also display chromosome instability, similar to those in PinX1-inhibited cells [82]. Thus, PinX1 mutant tumors are likely derived from multiple epithelial cells, presumably due to chromosome instability. These results are consistently previous findings that PinX1 potently controls tumorigenicity of cancer cells [66], and that telomerase overexpression promotes tumorigenesis, but is not as oncogenic as PinX1 knockout. Notably, telomerase activation and telomere lengthening induced by due to lack of PinX1 [82] and telomere dysfunction due to telomere shortening and deprotection share some common features including anaphase bridges, aneuploidy, translocation and chromosome instability both in MEFs and tumors. However, unlike the latter, the former induces chromosome instability in the presence of functional p53 and the absence of TIF (telomere dysfunction-induced focus) [82]. These results suggest that downregulation of PinX1 induces chromosome instability and tumorigenesis without activating DNA damage pathways [82].

## PINX1 IS AN ATTRACTIVE NEW TARGET IN CANCER THERAPY

An increasing body of evidence suggests that PinX1 is an attractive new target in cancer therapy. We have originally shown that whereas reducing PinX1 in human cancer cells increases their tumorigenicity, overexpression of PinX1, especially its small TID domain (telomerase-inhibitory domain) drives cancer cells into crisis and potently suppresses their ability to form tumors in mice [66]. These findings have now been confirmed and expanded to many different human cancer cells by transducing recombinant full length PinX1 protein or its protein fragments containing TID into cancer cells using HIV-Tat-mediated delivery [111] or mBAFF-targeted delivery [112]. Interestingly, overexpression of PinX1 also enhances the sensitivity of human cancer cells to chemotherapy drug 5-fluorouracil [113]. Furthermore, human PinX1 is located to 8p23 [13, 66], a frequent LOH region in common human epithelial cells [9, 10, 12, 15, 19, 21, 25, 26], and PinX1 protein is indeed downregulated commonly in many human epithelial cancers examined [13, 26, 66, 82]. Moreover, reducing PinX1 levels by heterozygous knockout or knockdown leads to chromosome instability and tumorigenesis in vitro and in vivo, with most tumors in PinX1+/- mice being carcinomas and sharing tissues of origin with human cancer types linked to 8p23 [82]. The therapeutic significance of these findings is further substantiated by the demonstration that the ability of PinX1 knockout or knockdown to induce chromosome instability is fully blocked by TERT knockout or knockdown [82]. Indeed, inhibition of telomerase by a wide range of approaches including chemical inhibitors or vaccines have been shown to potently inhibit cancer cell growth and even cancer stem cells in vitro, in vivo, with promising results in clinical trials [114, 115]. Therefore, these results together indicate that inhibition of telomerase by PinX1, especially its small telomerase inhibitory domain might be used to treat cancers that have telomerase activation.

## CONCLUSION AND FUTURE DIRECTIONS

Originally identified as a TRF1/Pin2-binding protein, PinX1 is a potent telomerase inhibitor. Recent results have not only demonstrated that PinX1 is essential for maintaining telomeres at the optimal length, but also discovered that Pinx1 is a sought-after major tumor suppressor at human chromosome 8p23 that is essential for maintaining chromosome stability in vitro and in vivo. Moreover, emerging evidence suggest that PinX1 and especially its small telomerase inhibitory domain might be a potential new drug target for treat cancers that have telomerase activation. However, many questions remained to be addressed including how PinX1 regulates chromosome stability and tumorigenesis, how PinX1 is regulated under physiological and pathological conditions, how to develop PinX1-based cancer therapy, and whether PinX1 has other new functions. Further studies on this relatively unknown protein would uncover novel insight into the regulation of chromosome stability and tumorigenesis and might eventually lead to new therapies for cancers.
